# Experimental Evidence of Mechanical Isotropy in Porcine Lung Parenchyma

**DOI:** 10.3390/ma8052454

**Published:** 2015-05-08

**Authors:** Benjamin Weed, Sourav Patnaik, Mary Rougeau-Browning, Bryn Brazile, Jun Liao, Raj Prabhu, Lakiesha N. Williams

**Affiliations:** 1 Department of Agricultural and Biological Engineering, Mississippi State University, Starkville, MS 39762, USA; E-Mails: benjaminweed@gmail.com (B.W.); sourav.s.patnaik@gmail.com (S.P.); blb285@gmail.com (B.B.); jliao@abe.msstate.edu (J.L.); rprabhu@abe.msstate.edu (R.P.); 2 Department of Genetics, University of Georgia, Athens, GA 30602, USA; E-Mail: mar3088@uga.edu

**Keywords:** pulmonary trauma, blast lung, lung parenchyma, mechanical isotropy

## Abstract

Pulmonary injuries are a major source of morbidity and mortality associated with trauma. Trauma includes injuries associated with accidents and falls as well as blast injuries caused by explosives. The prevalence and mortality of these injuries has made research of pulmonary injury a major priority. Lungs have a complex structure, with multiple types of tissues necessary to allow successful respiration. The soft, porous parenchyma is the component of the lung which contains the alveoli responsible for gas exchange. Parenchyma is also the portion which is most susceptible to traumatic injury. Finite element simulations are an important tool for studying traumatic injury to the human body. These simulations rely on material properties to accurately recreate real world mechanical behaviors. Previous studies have explored the mechanical properties of lung tissues, specifically parenchyma. These studies have assumed material isotropy but, to our knowledge, no study has thoroughly tested and quantified this assumption. This study presents a novel methodology for assessing isotropy in a tissue, and applies these methods to porcine lung parenchyma. Briefly, lung parenchyma samples were dissected so as to be aligned with one of the three anatomical planes, sagittal, frontal, and transverse, and then subjected to compressive mechanical testing. Stress-strain curves from these tests were statistically compared by a novel method for differences in stresses and strains at percentages of the curve. Histological samples aligned with the anatomical planes were also examined by qualitative and quantitative methods to determine any differences in the microstructural morphology. Our study showed significant evidence to support the hypothesis that lung parenchyma behaves isotropically.

## 1. Introduction

Pulmonary injury, including pulmonary contusion and pulmonary laceration, is a serious source of morbidity and mortality following blunt or blast trauma [1,2,3,4,5,6,7]. Common causes for civilians include motor vehicle accidents, falls, assaults, and sports injuries [8,9,10]. Additionally, explosions and other sources of blast trauma can cause severe pulmonary injuries, and are commonly seen in military or civilian victims of such events [1,2,3,11,12]. Blast lung injury is the primary cause of death in those who initially survive an explosion [12]. The prevalence of these types of injuries has made pulmonary injury a crucial area of trauma research.

Lungs have a complex structure, which can be summarized as a network of stiff airway tubes, bronchi and bronchioles, embedded in a soft and porous tissue, lung parenchyma. The lungs are enclosed by the thin pleural membrane and immersed in pleural fluid. The parenchyma contains the gas-exchanging alveoli which are very soft and highly susceptible to damage. Damage to the parenchyma leads to bleeding, edema, and collapse of the microstructure, which prevents gas exchange [13]. In serious cases this ultimately leads to the inability of the lungs to adequately oxygenate the blood and even medically supplemented oxygen may not be sufficient to prevent death [4]. The degree of lung damage a trauma victim has sustained is difficult to diagnose in a clinical setting. Physical experiments which could create a predictive index for these injuries involving human cadavers or live animals are costly and logistically difficult to perform. Finite element methods are a promising option for performing controlled experiments of different forms and severities of traumatic events.

Finite element methods are commonly used in research for simulating different mechanisms of injury [4,7,11,14,15]. These simulations require constitutive relationships to represent the mechanical properties of the materials. Previous studies have explored the mechanical responses of lung parenchyma under different loading conditions [16,17,18]. These studies have all assumed that lung parenchyma behaves isotropically, but to our knowledge, no study has verified this assumption. Moreover, reports that have addressed isotropy in other materials have used the comparison of values derived from stress-strain curves such as modulus, extensibility, or stress and strain at failure [19,20,21,22]. These methods of comparisons offer insight into the isotropy in a material, but do not compare the entire stress-strain relationships of different loading directions along quantifiable histological data, which may be useful for certain materials or loading multiaxial conditions.

In this study we evaluated lung tissue in compression with three experimental groups comprising specimens aligned with the three anatomical planes: sagittal, frontal, and coronal. We subjected these groups to uniaxial compression, with each group corresponding to one of the anatomical planes. Stress-strain curves of each group were generated from the test data, and the groups were compared to determine the differences among loading directions in the stress and strain values at 10% intervals of the curve. These differences were evaluated for statistical significance between direction groups. Furthermore, histological investigations (Movat, Massons) and analysis of the histological micrographs were used to determine if any morphological differences were apparent at the microstructure level.

## 2. Materials and Methods

### 2.1. Tissue Procurement and Preparation

**Figure 1:**
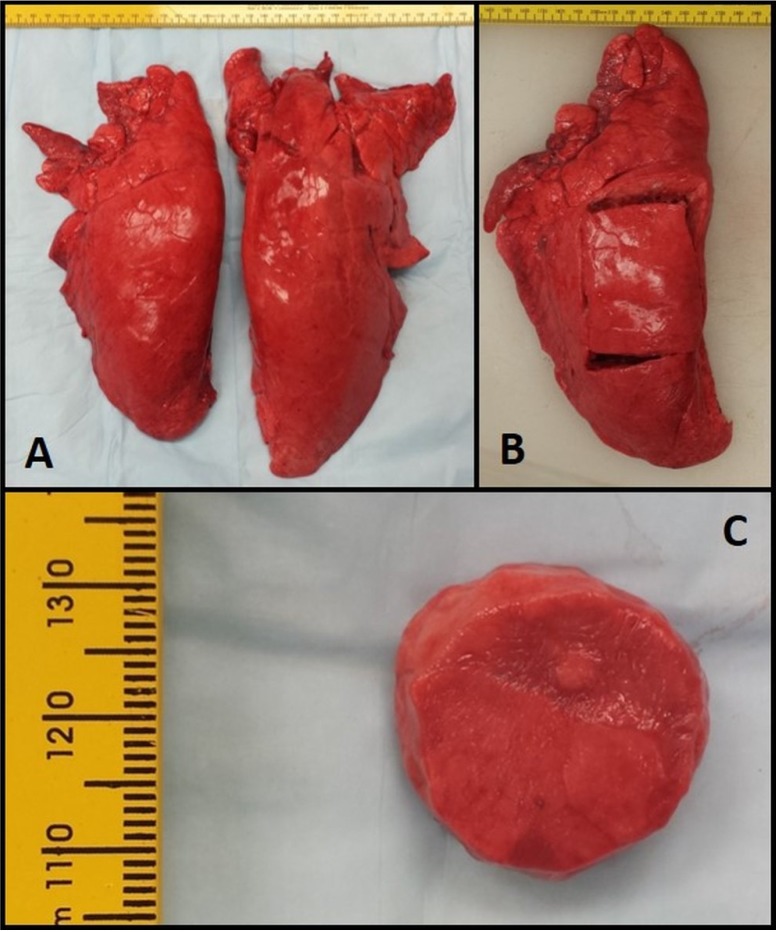
(**A**) Freshly obtained pig lung. (**B**) 60 mm × 100 mm rectangular sections were removed. (**C**) 10 mm thick cylindrical samples were prepared.

**Figure 2:**
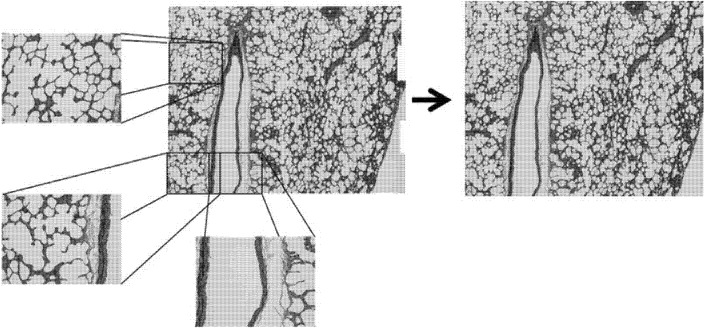
Composite micrograph composed of individual micrographs.

Porcine lungs were obtained from a local abattoir, in accordance with Mississippi State University Institutional Animal Care and Use Committee regulations. All specimens were obtained from market ready pigs of approximately 12 months of age weighing 225 pounds. Lungs were obtained immediately following sacrifice, placed in sealed plastic bags in a cooler on ice and transferred within thirty minutes to the laboratories in the Department of Agricultural and Biological Engineering at Mississippi State University for mechanical testing (Figure 1a). To alleviate the effects of post mortem tissue changes, all mechanical tests were performed within twelve h of sacrifice. Lungs were dissected of the pleural membranes and a section of the lung, approximately 60 mm by 100 mm rectangular sections were isolated from the center area (Figure 1b). Twenty mm cylindrical test specimens were dissected from the larger section of lung. These cylindrical samples were 10 mm thick (Figure 2c). The cylindrical samples were prepared such that they were aligned with one of the three anatomical planes; sagittal, frontal, and coronal. The three alignments were used as our three comparison groups.

### 2.2. Histological Preparation and Light Microscopy

Three specimens, one from each of the aforementioned anatomical planes, were prepared for histological examination. Specimens were fixed using 10% neutral buffered formalin, paraffin embedded, sectioned to 5 μm thickness, and mounted to glass slides. One set of slides was stained with Movat’s Pentachrome to observe the composition of structural proteins, and a second set of slides was stained with Masson’s Trichrome to observe the blood vessels and fibrous tissue. Prepared specimens were analyzed via light microscopy using a Leica DM2500 light microscope (Leica Microsystems, Wetzlar, Germany) at 100× magnification to observe any qualitative differences in microstructure.

Specimens were quantitatively analyzed for differences in microstructure using ImageJ (National Institute of Health, Bethesda, MD, USA). For each orientation, a series of twenty five overlapping light microscope images were obtained. These micrographs were compiled using the MosaicJ plugin (Ecole Polytechnique Federale De Lausanne, Lausanne, Switzerland) for ImageJ. Briefly, overlapping micrographs were positioned within the MosaicJ window and the software was used to create a single cohesive image based on overlapping portions of separate micrographs. This allowed for larger regions of the slide-mounted sample to be imaged without omitting portions of larger alveoli. The cohesive images were then cropped to create the largest rectangle available. Figure 2 illustrates how individual micrographs contributed to the composites, and how the uneven edges were cropped. The final composites for each direction were thresholded, inverted, and analyzed using the “Analyze Particles” feature in ImageJ. In this tissue the most relevant, and most readily observed by this method, feature is the alveolar spaces (white) of the lung tissue. “Analyze Particles” was configured to filter out particles with an area of less than 2000 pixels, approximately 2839 square microns, to eliminate extremely small particles or artifacts. Edge particles were also filtered. Roundness, Circularity, Solidity, and Aspect Ratio of the Fit Ellipse were plotted as histograms and analyzed to discern the 1st, 2nd and 3rd quartiles of the histograms to compare any differences among the alignment planes. These parameters describe particle shape and provide insight into particle elongation and tortuosity. Formulas for these parameters are shown in Appendix.

### 2.3. Compression Testing

**Figure 3:**
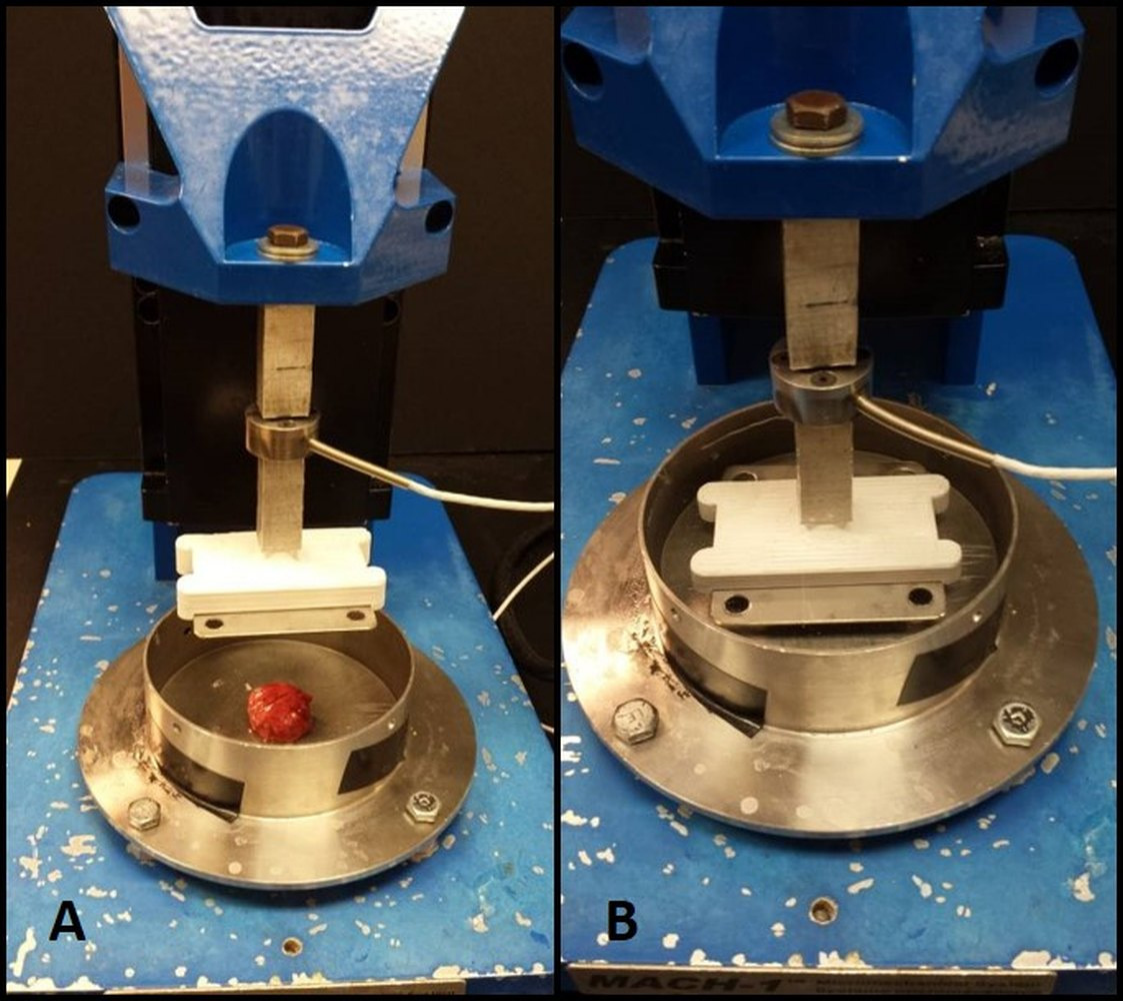
Mach1 Micromechanical Testing System^®^. (**A**) Sample in dish with compression platen removed; (**B**) Compression platen lowered onto sample in testing dish.

Specimens were subjected to uniaxial compressive loading using the Mach1 Micromechanical Testing System^®^ (Biomomentum, Laval, QC, Canada). A flat, disc-shaped metal platen was used for compression testing. The sample was attached with a small amount of high-viscosity cyanoacrylate ester adhesive (Permabond LLC, Pottstown, PA, USA) to prevent slipping of the sample from below the compression head, without introducing confinement effects that would compromise the uniform stress state assumption. Samples were submerged in Phosphate buffer saline (PBS) to simulate physiological conditions and prevent samples from drying during the test. Figure 3 shows the testing apparatus. The samples were pre-loaded to two grams-force, and pre-conditioned by cyclic loading to ten percent strain for ten cycles. We chose to precondition because lung is under constant cyclic loading during normal physiological conditions, and the preconditioning mimics this cycling. Specimens were then loaded to ten kilograms-force at a rate of ten percent strain (engineering) per second, with the loading velocity adjusted accordingly for each specimen. Load and displacement data were collected by the Mach1 software for further analysis.

## 3. Data Analysis

### 3.1. True Stress versus True Strain Conversions

Data from our mechanical tests were processed in Microsoft Excel (Microsoft, Redmond, WA, USA) to create true stress-true strain curves. Data from each test were converted to engineering stress and engineering strain using the following Equations (1) and (2) (Appendix). Data were further processed to true stress and true strain using Equations (3) and (4) (Appendix). These true stress-strain conversions assume a uniform stress state and incompressibility. These assumptions are discussed further in the limitations section of this manuscript.

### 3.2. Interpolation of True Stress-True Strain Curves

We observed the true stress and true strain at equivalent percentages of the stress-strain curve in order to compare tests within each anatomical plane and between anatomical planes. Because the sampling rate during testing yields results that do not coincide with even percentages, an Excel-based interpolation script was used to create an equivalent curve with 10 data points at multiples of 10% (10%, 20%, …, 100%) for each experimental data set. Briefly and for a given set of test data, this interpolation script determined the maximum true stress and true strain values and normalized all true stress and true strain values relative to the maximum, respectively. The distance was then determined between each recorded data point by using the Pythagorean distance formula. For each data point, the accumulated distance was divided by the total distance for the data set to determine the percentage of the total for that data point. Using these newly assigned percentages for each data point, the true stress-true strain data points closest to a 10% strain were then selected. A linear interpolation was done between these two points to create a new data point, which represented the 10% point of the experimentally recorded curves. This process was repeated for each multiple of 10% until 90% was reached. The final experimentally recorded data point was used as the 100% data point. This script was run for each experimental data set to generate a bank of curves that would allow for precise analysis.

### 3.3. Comparison of Recorded Data among Anatomical Planes

**Figure 4:**
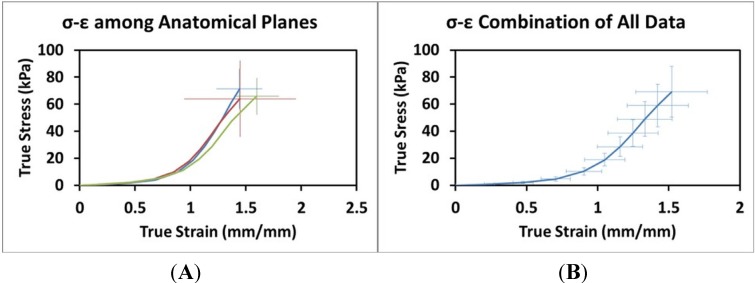
(**A**) Stress-Strain relationship across anatomical planes (Frontal (blue): *n* = 18, Sagittal (red): *n* = 13, Transverse: *n* = 12). (**B**) Combination of all Stress-Strain data (*n* = 43). Error bars indicate +/− one standard deviation.

The interpolated data sets from each experimental test were compiled in Excel and the data at increments of 10% strain within each anatomical plane were averaged to create a characteristic curve for each anatomical plane. The average of the characteristic curves were plotted with standard deviation (Figure 4).

Statistical analysis was performed to compare the differences in stress and strain in each of the three anatomical planes. At 10% strain increments, the stress-strain data were tested using a single-factor ANOVA with a 95% confidence interval to determine if there was a significant difference among each of the three anatomical planes. Statistics were performed using in Microsoft Excel.

## 4. Results

**Figure 5:**
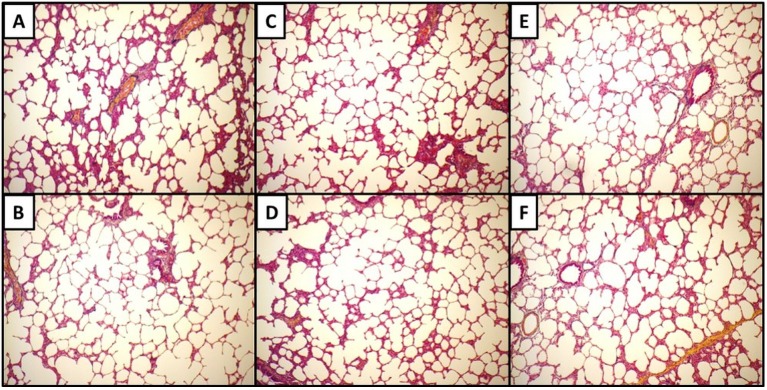
Movat’s Pentachrome for the sagittal (**A**,**B**), frontal (**C**,**D**) and transverse (**E**,**F**) planes.

**Figure 6:**
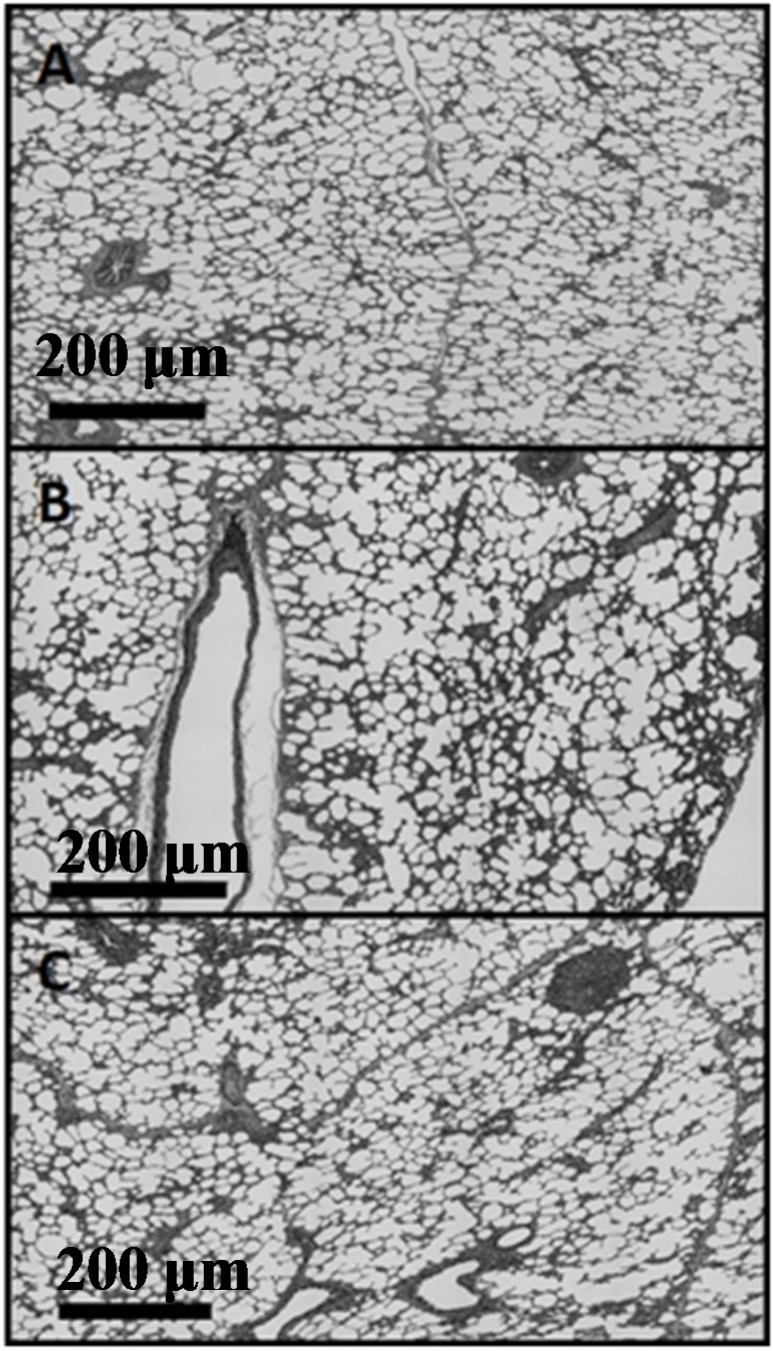
Composite micrographs of (**A**) saggital, (**B**) frontal, (**C**) transverse planes (200 μm scale).

**Figure 7:**
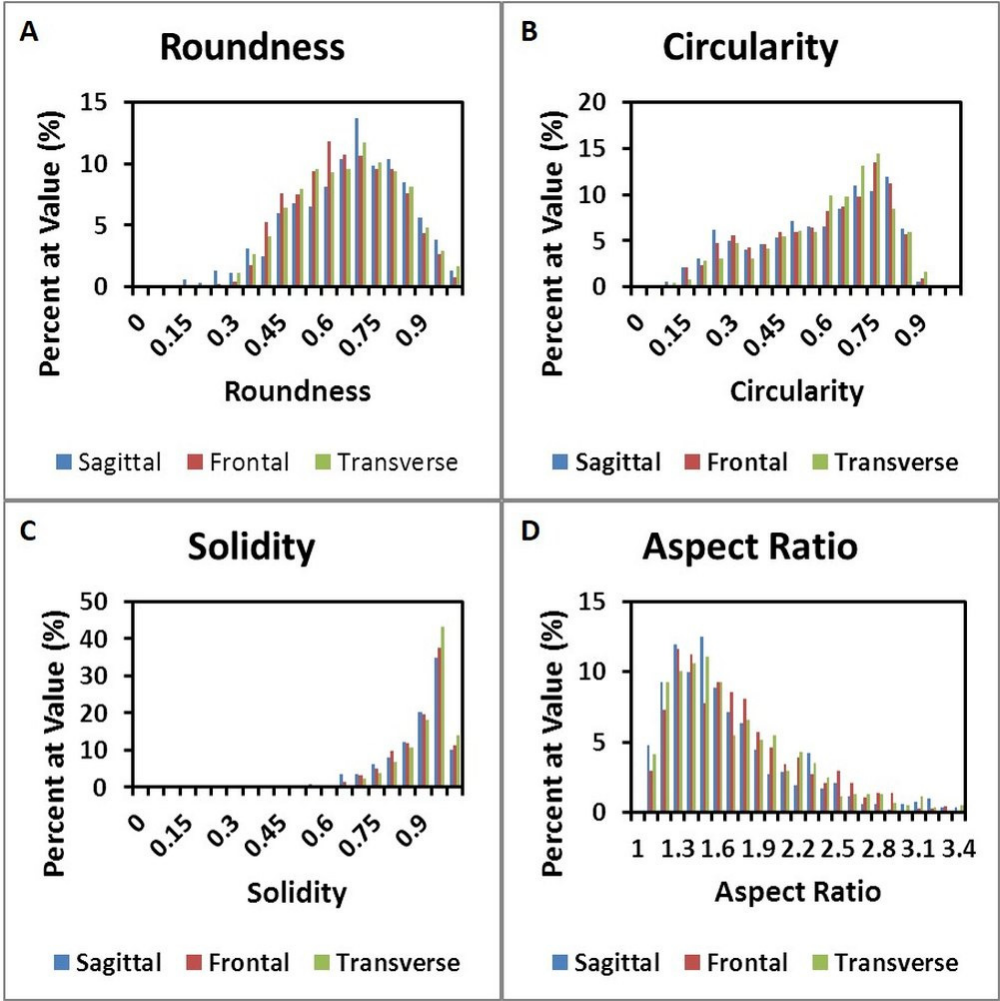
Histograms for (**A**) Roundness, (**B**) Circularity, and (**C**) Solidity. (**D**) shows the aspect ratio of samples.

Micrographs for each anatomical plane are presented in Figure 5. Figure 5A-B, C-D, and E-F show Movat’s Pentachrome for the sagittal, frontal, and transverse planes, respectively. These micrographs do not indicate any apparent qualitative differences in morphology or alignment of microstructural features among the three anatomical planes. Composite Micrographs created with MosaicJ are shown in Figure 6; Figure 6A is sagittal, Figure 6B is frontal, Figure 6C is transverse. The corresponding histograms for a particle analysis, particles being alveolar spaces, are shown in Figure 7. Histograms for Roundness (Figure 7A), Circularity (Figure 7B), and Solidity (Figure 7C), were prepared with bins from 0 to 1, incremented by 0.05, where the columns indicate the percentage of particles that fell into each bin. The aspect ratio (Figure 7D), was prepared with bins from 1.0 to 3.4, incremented by 0.1, and columns denoting the percentage of particles for each bin. It should be noted that some aspect ratio data was not displayed in this histogram in the interest of more effectively highlighting the majority of the data; 3.4 was chosen as the end of the axis because this was the final value at which more than two particles occurred within a single bin for any of the three alignments. Table 1 shows the 1st quartile, median, and 3rd quartiles for the histograms. Histograms of particle analysis parameters had little difference among the different orientation planes.

**Table 1:** The 1st quartile, median, and 3rd quartiles for the histograms.

**Roundness**	**Sagittal**	**Frontal**	**Transverse**
1st Quartile	0.53375	0.512	0.512
Median	0.66	0.626	0.647
3rd Quartile	0.77225	0.75	0.761
**Circularity**	**Sagittal**	**Frontal**	**Transverse**
1st Quartile	0.385	0.411	0.451
Median	0.5855	0.6	0.618
3rd Quartile	0.72475	0.722	0.723
**Solidi**	**Sagittal**	**Frontal**	**Transverse**
1st Quartile	0.812	0.824	0.853
Median	0.889	0.898	0.912
3rd Quartile	0.93525	0.936	0.94
**Aspect Ratio**	**Sagittal**	**Frontal**	**Transverse**
1st Quartile	1.29475	1.333	1.314
Median	1.515	1.598	1.546
3rd Quartile	1.87475	1.953	1.955

True stress-true strain curves for each anatomical plane are shown in Figure 4A. The composite curve for the average of the three planes is shown in Figure 4B. The results of the statistical comparisons for each percentage are presented in Table 2 and Table 3. There were no significant differences in either stress or strain among the three anatomical planes for any percentage level.

**Table 2:** Results of statistical comparisons for the samples.

Path %	Frontal	Sagittal	Transverse	*F*	*p*-value	Frontal	Sagittal	Transverse	*F*	*P*-value
	Avg. True σ	Avg. True σ	Avg. True σ			Avg. True ε	Avg. True ε	Avg. True ε		
10	619.39	729.75	957.42	2.6893	0.0793	0.2293	0.2251	0.2477	0.0470	0.9542
20	1,588.29	1,934.04	2,390.74	2.5329	0.0912	0.4580	0.4488	0.4937	0.0468	0.9544
30	3,984.76	4,568.52	5,304.14	1.4654	0.2423	0.6817	0.6642	0.7301	0.0462	0.9549
40	9,563.28	9,819.38	10,977.00	0.3470	0.7087	0.8778	0.8506	0.9318	0.0453	0.9557
50	18,377.42	17,719.62	19,178.35	0.0115	0.9886	1.0198	0.9872	1.0789	0.0448	0.9562
60	28,466.14	26,711.31	28,380.77	0.0223	0.9780	1.1261	1.0896	1.1895	0.0444	0.9566
70	39,023.73	36,054.37	37,928.60	0.0771	0.9259	1.2130	1.1766	1.2817	0.0442	0.9568
80	49,760.69	45,514.93	47,479.43	0.1367	0.8726	1.2907	1.2584	1.3727	0.0443	0.9567
90	60,601.53	54,822.69	56,676.20	0.2193	0.8040	1.3625	1.3489	1.4820	0.0453	0.9558
100	71,323.68	64,005.78	65,778.25	0.2841	0.7541	1.4406	1.4472	1.5977	0.0461	0.9550

**Table 3:** Standard deviations for statistical comparisons of the samples. Dev.: Deviations.

Part %	Frontal		Sagittal		Transverse	
	Strain Dev.	Stress Dev.	Strain Dev.	Stress Dev.	Strain Dev.	Stress Dev.
10	0.030915423	127.2781798	0.075739326	240.5005433	0.027138761	331.5140315
20	0.061629963	347.8942716	0.150734704	648.4193448	0.054122752	817.186667
30	0.090535682	829.7268617	0.221484316	1556.705447	0.078778275	1624.193247
40	0.110923627	1890.534598	0.280026781	3438.735718	0.097867071	2602.47132
50	0.123904198	3539.070152	0.32436637	6711.154692	0.113630437	3650.112918
60	0.137258653	5585.462839	0.359870173	10716.82231	0.127062902	5247.527794
70	0.150673246	7795.522934	0.392218749	14986.77743	0.139112415	7176.190338
80	0.164835636	10072.46936	0.424828203	19386.53237	0.151445696	9218.119181
90	0.180556847	12424.48858	0.46166626	23807.36975	0.168660868	11333.30934
100	0.208370407	14937.09112	0.504503056	28290.23423	0.198543701	13672.60307

## 5. Discussion

True stress-true strain curves for each anatomical plane appear to be similar, and the error bars indicate the variation among specimens is much greater than the variation between the averaged curves for each anatomical plane. It is well known that biological tissues exhibit a large degree of inter-specimen variation, and the degree to which the plane curves vary appears to be within one standard deviation for each other curve, respectively. This observation, while qualitative, strongly supports our hypothesis of isotropy. Statistical comparison of the stress and strain values for each multiple of 10% indicates no significant differences between any of the three planes or the average of the three planes. This quantitative observation also supports our hypothesis of isotropy.

The interpolation method presented was formulated with the interest of comparing curves effectively. The method for determining the percentages of the curve was chosen as a means of giving equivalent weight to both stress and strain. We believed this approach to be preferable to basing percentages on only one parameter because the characteristic curves for biological materials, which are often described as hyperelastic, are commonly observed to have two distinct regions of tissue behavior. The first region is described as the toe-in region and is dominated by the realignment of tissue structures to resist loading. The second region is described as the linear region and is dominated by significant resistance to loading as the tissue builds towards failure. A comparison based on strain percentages would capture more of the toe-in region and less of the linear region, essentially hiding a large amount of tissue behavior in the higher strain percentages. Likewise, a percentage comparison based on stress would capture more of the linear region and less of the toe-in, hiding a large amount of tissue behavior in the lower stress values. The combined weight of the two parameters allows the entire stress-strain path to be considered equally.

Quantitative analysis of micrographs did not indicate apparent differences in the morphology of porcine lungs (Figure 7 and Table 1). The quantitative particle analysis, which showed the morphological parameters had very similar values and distributions among the orientation planes, complimented the qualitative observations. Overall, the histological examinations supported the hypothesis that porcine lung is an isotropic material. Analysis of microstructural features is an important part of understanding the hierarchical organization of biological tissues. The change in organization, shape, and size of some features under deformation lends insight to possible physiological changes in the biological system. Microstructural changes during loading can be used to understand the sub structural evolution of lung, or other biological tissues, under loading and thus aid in modeling complex tissue behaviors. The data presented here strongly supports the widely held belief that lung parenchyma is an isotropic material. Despite this belief, we are not aware of a thorough analysis of this assumption. Beyond the implications for lung tissue, we believe the methods presented here represent a strong system for assessing isotropy in other tissues. This is valuable for future research as biological tissues may be isotropic or anisotropic, and a confident understanding of a given tissue’s behavior is crucial for effective constitutive modeling. Additionally, verification of isotropy allows the analysis of more complex material properties such as viscoelasticity, strain rate dependency, and stress state dependency without considering the complexity of anisotropy. The ultimate result is a more efficient path towards a complete understanding of the material properties of lung, and other biological tissues of interest to the research community.

## 6. Conclusions

This study presents a novel method for testing mechanical isotropy in biological tissues. This method was used to demonstrate the isotropic nature of porcine lung parenchyma. This was a widely used assumption in the body of literature, but had not been explicitly analyzed or proven. This is significant for soft-tissue biomechanics in general, as it presents a means for testing isotropy in other tissues. It is significant for lung biomechanics research because it allows for future research to be conducted without concern for overlooking anisotropy in more complex material properties. Future studies will address important behaviors such as viscoelasticity, strain rate dependency, stress-state dependency, and microstructural evolution of lung parenchyma.

## 7. Limitations

The conversion formulas for true stress and true strain rely on assumptions of uniform stress state and incompressibility. Our mechanical testing setup is intended to provide the most uniform stress state possible, but there may be some uneven distributions that limit this aspect of the assumption. Moreover, the assumption of incompressibility is inexact, as the lung contains solid and gas components, which have a high probability of being compressible. Considering that our test procedure does not actively track the changing cross-sectional area it is necessary to use some form of conversion to achieve true stress, as this method was used in our previous publication [23].

The interpolation method described uses a linear interpolation process, which slightly overestimates the interpolated values given that our curves are concave upward. Future studies will explore the incorporation of Newton-Raphson methodology to alleviate this limitation.

## Appendix

Formulas for Stress-Strain Calculations
  1. ${\text{σ}}_{engineering}=\frac{force}{area_{undeformed}}$
  2. ${\text{ε}}_{engineering}=\frac{length_{undeformed}-displacement}{length_{undeformed}}$
  3. ${\text{σ}}_{true}={\text{σ}}_{engineering}*\left(1+{\text{ε}}_{engineering}\right)$
  4. ${\text{ε}}_{true}=\ln\left(1+{\text{ε}}_{engineering}\right)$
Formulas for Shape Descriptors Used in Particle Analysis
  1. $Roudness=4\times \left(\frac{Area}{\text{π}\;\times \;{\left(Major\;Axis\;of\;Fit\;Ellipse\right)}^{2}}\right)$
  2. $Circularity=4\text{π}\times \left(\frac{Area}{Perimeter^{2}}\right)$
  3. $Solidity=\frac{Area}{Convex\;Area}$
  4. $Aspect\;Ratio=\frac{Major\;Axis\;of\;Fit\;Ellipse}{Minor\;Axis\;of\;Fit\;Ellipse}$
